# External validation of anastomotic leakage risk analysis system in patients who underwent colorectal resection

**DOI:** 10.3906/sag-1807-205

**Published:** 2019-02-11

**Authors:** Sadettin ER, Sabri ÖZDEN, Faruk KOCA, Barış Doğu YILDIZ, Bülent Cavit YÜKSEL, Mesut TEZ

**Affiliations:** 1 Department of General Surgery, Numune Training and Research Hospital, Ankara Turkey

**Keywords:** Anastomotic leakages, validation studies, colorectal carcinoma

## Abstract

**Background/aim:**

One of the most feared complications after colon resection for carcinoma is anastomotic leakage. Prediction of anastomotic leakage can alter pre- and perioperative management of patients. This study validates an anastomotic leakage prediction system.

**Materials and methods:**

Ninety-five patients who underwent colonic resection between 1 January 2016 and 30 January 2017 were included in the study. Patient records and electronic charting system data were used to calculate anastomotic leakage risk on the http://www.anastomoticleak.com/ website.

**Results:**

Fifty-six (58.9%) patients were male and thirty-nine (41.1%) were female. The mean age was 61.7 (min: 33, max: 90). Six (6.3%) patients had anastomotic leakage. According to the ROC analysis, the area under curve for the prediction system was 0.767.

**Conclusion:**

The prediction system for anastomotic leakage produced significant results for our patient population. It can be effectively utilized in preoperative and perioperative measures to prevent anastomotic leakage.

## 1. Introduction

Despite the advances in surgical techniques, anastomotic leakage (AL) in colorectal surgery remains an important problem. Mortality in patients with AL can be as high as 39% secondary to sepsis and generalized peritonitis (1). The incidence of AL varies between 1% and 39% in different studies (2). There is a relationship between local and distant cancer recurrence in colorectal cancer patients with AL (3). There are numerous factors influencing AL, which makes it necessary to undertake prediction based on accurate criteria (4,5). 

Before a prospective study enrolling 3000 patients evaluating risk factors for AL by Frasson et al. (6), the absence of a simple nomogram for the prediction of AL was a problem. Frasson et al. devised a nomogram from the multivariate analysis of their data on preoperative serum total proteins, male sex, ongoing anticoagulant treatment, intraoperative complication, and number of hospital beds (6). The current study is an external validation of this AL risk analysis system proposed by Frasson et al.

## 2. Materials and methods

### 2.1. Patient selection

Ninety-five patients who underwent colonic resection (either elective or emergent) between 1 January 2016 and 30 January 2017 were included in the study. The protocol of this study was approved by the local ethics committee (Approval No: E-17-390). A total of 148 patients were identified. The exclusion criteria were the history of end colostomy, protective ileostomy or colostomy, R2 resection, and the presence of mucous fistula. The patient records and electronic charting system data were used to calculate the anastomotic leakage risk on the website using data concerning sex, body mass index (BMI), preoperative serum total proteins, ongoing anticoagulant treatment, intraoperative complications, and number of hospital beds. This risk calculator is available online at http://www.anastomoticleak.com/. This nomogram emerged from Frasson et al.’s study, which was published in August 2015 (6).

### 2.2. Statistical analysis

The continuous variables were evaluated using the Kolmogorov–Smirnov test. The Mann–Whitney U and chi-square tests were used to compare the parametric and nonparametric variables. The receiver operating characteristic curve (ROC) was employed where appropriate. P < 0.05 was accepted as significant.

## 3. Results

Fifty-six patients (58.9%) were male and thirty-nine were female (41.1%). The mean age was 61.7 years (range: 33–90). Six patients (6.3%) had AL. The data on these six patients are presented in Table 1. The risk score assessment of these patients revealed a statistically significant difference between those who had AL and those who did not (Table 2). When the type of surgery was analyzed, it was seen that the patients who underwent low or very low anterior resection had higher incidences of AL (n = 5). The remaining one patient had anterior resection. According to the ROC analysis for AL, the area under the curve was 0.767 (95% confidence interval, 0.485–1.000; P < 0.029) (Figure).

**Table 1:** Data on anastomotic leakage scores and types of surgery.

	Anastomotic leakage			
	Present		Absent	
	n	%	n	%
Sex (M/F)	5/1	8.9/2.6 (%)	51/38	91.1/ 97.4(%)
Age (mean ± SD) (min–max)	56.67 ± 11.40 (45–77)		62.09 ± 11.47 (33–90)	
Obesity (BMI > 30 kg/m2) Yes	2	25 (%)	6	75 (%)
No	4	4.6 (%)	83	95.4 (%)
Oral anticoagulation				
Present	1	9.1 (%)	10	90.9 (%)
Absent	5	6 (%)	79	94 (%)
Intraoperative complication				
Present	0	0 (%)	0	0 (%)
Absent	6	6.3 (%)	89	93.7 (%)
Type of surgery				
OAR	1	0 (%)	7	7.9 (%)
OERH	0	0 (%)	4	4.5 (%)
LAR	0	16.7 (%)	1	1.1 (%)
LELH	0	0 (%)	1	1.1 (%)
LERH	0	0 (%)	3	3.4 (%)
LLH	0	0 (%)	2	2.2 (%)
LLAR	1	16.7 (%)	18	20.2 (%)
LRH	0	0 (%)	7	7.9 (%)
OLH	0	0 (%)	9	10.1 (%)
OLAR	0	0 (%)	6	6.7 (%)
LVLAR	4	66.7 (%)	10	11.2 (%)
OSH	0	0 (%)	17	19.1 (%)
TC	0	0 (%)	1	1.1 (%)
OVLAR	0	0 (%)	2	2.2 (%)
RHCO	0	0 (%)	1	1.1 (%)

OAR: Open anterior resection. OERH: Open extended right hemicolectomy. LAR: Low anterior resection. LELH: Laparoscopic extended left hemicolectomy. LERH: Laparoscopic extended right hemicolectomy. LLH: Laparoscopic left hemicolectomy. LLAR: Laparoscopic low anterior resection. LRH: Laparoscopic right hemicolectomy. OLH: Open left hemicolectomy. OLAR: Open low anterior resection. LVLAR: Laparoscopic very low anterior resection. OSH: Open right hemicolectomy. TC: Total colectomy. OVLAR: Open very low anterior resection. RHCO: Laparoscopic right hemicolectomy converted to open surgery. AL: Anastomotic leakage. BMI: Body mass index. SD: Standard deviation.

**Table 2:** Analysis of the AL scores.

	Mean	Standard deviation	Minimum	Maximum	P-value
AL risk score Present Absent	12.83 7.01	6.24 3.09	5 5	20 17	<0.05*

AL: Anastomotic leakage.

**Figure 1:**
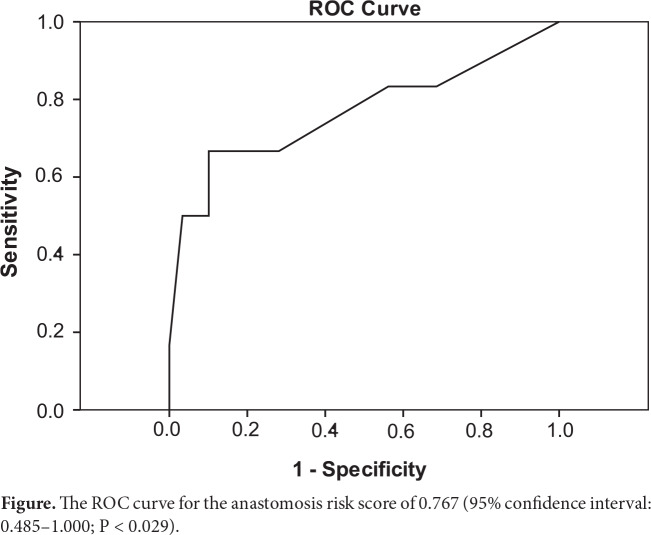
The ROC curve for the anastomosis risk score of 0.767 (95% confidence interval: 0.485–1.000; P < 0.029).

## 4. Discussion

The level of anastomosis is regarded as an important factor for AL. In the current study, AL was present at higher incidences in the patients who underwent low or very low anterior resection. In their randomized controlled trial, Rose et al. (7) showed that the AL rate was 16.8% in patients who had undergone laparoscopic colorectal surgery within 10 cm lower than the anal verge. 

AL can be seen in colorectal surgical procedures for both benign and malignant conditions. It can present in a wide spectrum between simple clinical symptomatology and death. The incidence of AL is reported to vary between 1% and 39% in different studies (2,8). The AL rate in our study (6.3%) was within this range. The prediction of AL is of paramount importance for the proper management of patients to minimize morbidity, mortality, hospital stay, and cost.

Fouda et al. (9) and Bokey et al. (10) found the mortality rate after AL as 32% and 10%–15%, respectively. AL is considered as an independent risk factor for local recurrence and survival (11). The lack of a standardized prediction and diagnosis of AL allows this condition to maintain its hazardous course. 

In different studies, AL was identified based on abscesses, peritonitis, and systemic sepsis at 7 to 12 days postoperatively (12,13). In the current study, AL was diagnosed within 6 to 7 days after surgery.

A few studies investigated the early prediction of AL. According to our clinical experience, abdominal pain, purulent/intestinal discharge from drains or wounds, systemic fever, vomiting, abdominal distension, tachycardia, respiratory distress, and mental state changes suggest AL. However, these are usually seen in late stages of AL. In this context, Alves et al. (14) found that the absence of bowel movements for more than 4 days was related to AL. Another study reported that the presence of more than two comorbidities was an independent risk factor for AL (15). 

Lipska et al. reported that male sex had a relative risk of 2.3 (16). In our study, the male to female ratio was 5 to 1 for patients with AL. Although intraoperative complications were previously shown to be independent risk factors for AL (2), none of our patients had any adverse event perioperatively. Finally, increased BMI was found to increase AL in several studies (17,18), although visceral fat density was identified to be more sensitive than BMI (19). In our study, two (%33) of the six patients with AL had a BMI greater than 30.

There are a number of scoring systems in the medical literature; e.g., the colon leakage score (20) helps surgeons decide whether to create a protective stoma. Another scoring system, the modified Dutch Leak Score (DULK), has the four components of respiratory rate (>20/min), clinical deterioration, abdominal pain, and seroreactive protein (>250 mg/L). The predictive strength of the modified DULK was reported to be 17%–20% (21,22). The reason why we chose to validate the AL prediction system of Frasson et al. was because this scoring system has more objective parameters compared to the modified DULK score, such as sex, BMI, use of anticoagulants, intraoperative complications, serum protein level, and hospital size.

The mentioned scoring system cannot help to prevent AL, but it may help inform patients about the possible risk of AL. In conclusion, the AL prediction scoring system devised by Frasson et al. is simple to use and was applicable to our patient population. The early prediction of AL can assist in the management of AL patients, thus decreasing morbidity and mortality.

## References

[ref0] (1997). A multivariate analysis of factors contributing to leakage of intestinal anastomoses. J Am Coll Surg.

[ref1] (2004). Risk factors for anastomotic leakage after anterior resection of the rectum. Colorectal Dis.

[ref2] (2015). Anastomotic leak increases distant recurrence and long-term mortality after curative resection for colonic cancer. Ann Surg.

[ref3] (2009). Surgeons lack predictive accuracy for anastomotic leakage in gastrointestinal surgery. Int J Colorectal Dis.

[ref4] (2002). Colorectal surgery and anastomotic leakage. Dig Surg.

[ref5] (2015). Risk factors for anastomotic leak after colon resection for cancer: multivariate analysis and nomogram from a multicentric, prospective, national study with 3193 patients. Ann Surg.

[ref6] (2004). Complications in laparoscopic colorectal surgery: results of a multicentre trial. Tech Coloproctol.

[ref7] (2016). Minimally invasive management of anastomotic leaks in colorectal surgery. World J Gastrointest Surg.

[ref8] (2011). Early detection of anastomotic leakage after elective low anterior resection. J Gastrointest Surg.

[ref9] (1995). Postoperative morbidity and mortality following resection of the colon and rectum for cancer. Dis Colon Rectum.

[ref10] (2002). Factors associated with clinically significant anastomotic leakage after large bowel resection: multivariate analysis of 707 patients. World J Surg.

[ref11] (2007). Anastomotic leaks after intestinal anastomosis: it’s later than you think. Annals of Surgery.

[ref12] (2008). The management and outcome of anastomotic leaks in colorectal surgery. Colorectal Dis.

[ref13] (1999). Management of anastomotic leakage after nondiverted large bowel resection. J Am Coll Surg.

[ref14] (2003). Risk factors for anastomotic leakage after left-sided colorectal resection with rectal anastomosis. Dis Colon Rectum.

[ref15] (2006). Anastomotic leakage after lower gastrointestinal anastomosis: men are at a higher risk. ANZ Journal of Surgery.

[ref16] (2011). Risk factors for morbidity and mortality after single-layer continuous suture for ileocolonic anastomosis. Int J Colorectal Dis.

[ref17] (2003). Laparoscopic colectomy in obese and nonobese patients. J Gastrointest Surg.

[ref18] (2014). The impact of visceral obesity on surgical outcomes of laparoscopic surgery for colon cancer. Int J Colorectal Dis.

[ref19] (2011). Predicting the risk of anastomotic leakage in left-sided colorectal surgery using a colon leakage score. Journal of Surgical Research.

[ref20] (2009). Improved diagnosis and treatment of anastomotic leakage after colorectal surgery. Eur J Surg Oncol.

[ref21] (2013). The DULK (Dutch leakage) and modified DULK score compared: actively seek the leak. Colorectal Dis.

